# Overexpression of FES might inhibit cell proliferation, migration, and invasion of osteosarcoma cells

**DOI:** 10.1186/s12935-020-01181-3

**Published:** 2020-03-30

**Authors:** Yang Zhao, Zhimeng Wang, Qian Wang, Liang Sun, Ming Li, Cheng Ren, Hanzhong Xue, Zhong Li, Kun Zhang, Dingjun Hao, Na Yang, Zhe Song, Teng Ma, Yao Lu

**Affiliations:** 1 Department of Orthopaedic Surgery, Honghui Hospital, Xi’an Jiaotong University, No. 555 Youyi East Road, Xi’an, 710054 Shaanxi China; 2 Xi’an Medical University, Beilin District, Xi’an, 710054 Shaanxi China

**Keywords:** Osteosarcoma, Risk model, Prognosis, FES overexpression, Tumor suppressor

## Abstract

**Background:**

This study aimed to screen osteosarcoma (OS) prognosis relevant genes for methylation dysregulation, and the functional mechanisms of FES overexpression in OS cells were investigated.

**Methods:**

The OS prognosis relevant genes with differentially methylated positions (DMPs) identified from the GSE36001 and GSE36002 datasets, and the UCSC database, were used as a training set to construct a risk model, while the GSE21257 dataset was used as validation set. The expression levels of several key genes in OS cells after 5-Aza-2′-deoxycytidine treatment were detected by qPCR. The effects of FES overexpression on cell proliferation, cell cycle, migration, and invasion of MNNG/HOS were analyzed by CCK8, flow cytometry, and Transwell assays.

**Results:**

A total of 31 candidate genes, corresponding to 36 DMPs, were identified as OS prognosis relevant genes; from these, the top 10 genes were used to construct a risk model. Following validation of the risk model, FES, LYL1, MAP4K1, RIPK3, SLC15A3, and STAT3 showed expression changes between the OS and control samples. qPCR results showed that the expression of FES was significantly downregulated in three OS cell lines and increased after 5-Aza-DC treatment. The proliferation, cell cycle progression, migration, and invasion of MNNG/HOS cells were significantly inhibited after transfection with FES overexpression plasmid, and the protein expression of FYN and β catenin were decreased in MNNG/HOS cells by FES overexpression.

**Conclusions:**

The decrease in FES by hypermethylation was associated with OS prognosis, and might contribute to the proliferation, migration, and invasion of OS cells. FES, and its upstream FYN and β catenin, might coordinately exert a tumor suppressor effect in OS cells.

## Background

Osteosarcoma (OS) is a type of bone malignancy. Most diagnoses occur in children and adolescents, with a higher incidence males [1]. It is the sixth most common cancer in children and accounts for up about 3% of pediatric tumors worldwide [2]. OS is characterized by the formation of vast immature bone or osteoid tissue, and is derived from mesenchymal stem cells or progenitor cells of osteoblast lineage [3]. Surgical resection followed by adjuvant chemotherapy is a typical method of OS treatment. Although many advancements have been made in OS treatment, the 5-year survival rate of OS patient with high metastases is still low [3, 4]. Thus, early diagnosis is needed to prevent OS progression and improve the survival rate.

Recently, gene methylation, an important component of epigenetics research, has been implicated the development, progression, and prognosis of OS [5, 6]. Oh et al. [7] demonstrated a close correlation between hypermethylation and poor survival in OS. A significant decrease in the levels of SFRP2 mRNA and protein, induced by hypermethylation, have been detected in OS cells, which promotes OS invasion through activating Wnt signaling [8]. Additionally, HOTAIR that inhibits the methylation level of CDKN2A, is reported to regulate OS cell viability through the HOTAIR-miR126-DNMT1-CDKN2A axis [9].

Methylation induced gene silencing is an epigenetic mechanism associated with the development and progression of cancers [10, 11]. With the development of gene chip technology, abnormal mRNA and methylation expression data in cancer and normal samples, detected by gene chips, can be fully revealed. In the present study, an integrated analysis of OS mRNA and methylation data was performed, to identify OS prognosis relevant genes with differentially methylated positions (DMPs), and a risk model was constructed and validated. In addition, the expression levels of several key genes in three OS and normal osteoblast cell lines were detected. Genes with significant expression changes after 5-Aza-2′-deoxycytidine (5-Aza-dC) treatment were selected for further functional study, including proliferation, cell cycle, migration, and invasion assays. This study proposes a risk model for OS prognosis prediction and potential treatment targets for OS.

## Methods

### Data sources

A total of three OS datasets (GSE36001, GSE36002, and GSE21257), downloaded from the GEO database, were utilized. A set of 19 OS cell lines and six normal samples with both methylation expression data (GSE36002) and mRNA expression data (GSE36001) were examined in this study. Methylation data were produced using the Illumina HumanMethylation27 BeadChip platform (GPL8490), while the mRNA data were generated using the Illumina Human-6 v2.0 expression BeadChip platform (GPL6102). In addition, the clinical information and mRNA-Seq data of 259 sarcomas samples were downloaded from the UCSC database (http://xena.ucsc.edu/). The data from GSE36001, GSE36002, and the UCSC database were used as the training set, while the GSE21257 dataset was used as the validation set. The mRNA data and survival information of 53 OS patients were downloaded. GSE21257 data was obtained using the Illumina Human-6 v2.0 Expression BeadChip platform (GPL10295).

### DMPs analysis

Before data preprocessing, the methylation signals and unmethylated signals provided in GSE36002 were extracted firstly. The β value [methylation signal/(methylation signal + unmethylated signal + 100)] was calculated to indicate the methylation degree of one site. Following probe filtration, by deleting the probes with a p-value > 0.05 and located on sex chromosomes [12], a Bayesian method was utilized to conduct differential methylation analysis using the Limma (version 3.10.3) package [13]. Then, the Benjamini and Hochberg method was applied to adjust the p-value [14], and DMPs were screened with the cutoff of adjusted p-value < 0.05 and log_2_| fold change (FC)| > 0.585.

### Correlation analysis between methylation and mRNA data

Before conducting correlation analysis, gene expression values were obtained. In brief, according to the annotations file, the mRNA probes in the GSE36001 dataset were annotated in corresponding genes. If several probes were annotated in one gene, the mean expression value of these probes was considered as the expression value of this gene. Then, the Pearson’s correlation coefficient (r) between the screened DMPs from GSE36002 and their corresponding genes from GSE36001 was calculated. The genes with r < 0 and p-value < 0.05 were considered candidate genes for further analysis.

### Screening of prognosis relevant genes

The expression values of candidate genes and prognosis relevant clinical information of 259 sarcoma samples were extracted from the UCSC database, which included the data of overall survival and survival status. Then, univariate Cox regression analysis was conducted to screen candidate genes under the cutoff of p < 0.05.

### Risk model construction and validation

The top 10 screened candidate genes, listed by p-value, were considered as the preliminary range for risk score calculation. Based on the β and expression values of genes, the risk score for each sample was calculated by the following equation [15]:

1 $${\text{Risk score}} = \beta_{\text{gene 1}} *{\text{expr}}_{\text{gene 1}} + \beta_{\text{gene 2}} *{\text{expr}}_{\text{gene 2}} + \cdots + \beta_{\text{gene n}} *{\text{expr}}_{\text{gene n}}$$

In this formula, *β* indicates the prognostic correlation coefficient, and *expr* indicates the expression value of the gene. By setting median risk score as the boundary, the samples were divided into high and low risk types. The top 10 genes, based on their p-values (from small to large), were added into Eq. 1, one by one, until the risk score model could distinguish the maximum significant correlation between survival, and high and low risk samples. Finally, these genes were selected as key genes for risk model construction.

The expression data and corresponding survival information from the GSE21257 dataset was used as validation data. The process was repeated, followed by the extraction of the relative data for key genes. The genes with consistent expression trends in both the training and validation sets were selected for further analysis.

### Kaplan–Meier (KM) survival analysis

Following the calculation of the corresponding prognostic values of the key genes, the risk score of each sample was obtained using Eq. 1. Then, using the median risk score as the boundary, samples were divided into high risk and low risk groups, and KM survival analysis was conducted.

### Cells and cell culture

Three OS cell lines (Saos2, MG-63, and MNNG/HOS), and a normal human osteoblastic cell line, hFOB 1.19, were purchased from the Cell Collection of the Chinese Academy of Science (Shanghai, China). The Saos2 cells were maintained in McCoy’s 5A medium supplemented with 15% fetal bovine serum (FBS) and 1% penicillin–streptomycin (PS; BS734). The MG-63 and MNNG/HOS cell lines were cultured in MEM medium supplemented with 10% FBS and 1% PS, while the hFOB 1.19 cells were cultured in D-MEM/F-12 medium supplemented with 10% FBS and 1% PS. The three OS cell lines were incubated in humidified air at 37 °C with 5% CO_2_, while hFOB 1.19 cells were cultured at 37 °C with 5% CO_2_.

### 5-Aza-dC treatment

Approximately 3 × 10^6^ OS cells of the different lines were seeded into 6-well culture plates, respectively, followed by digestion with trypsin. After these cells were treated with 5 μM 5-Aza-DC (A3656, Sigma) for 48 h, the mRNA expressions levels of several key genes were detected by RT-PCR.

### Quantitative polymerase chain reaction (qPCR)

Total RNA from each cell line was extracted using TRIzol reagent (Cat. no. 9109; Takara, Japan) based on the manufacturer’s instructions. The expression levels of several key genes used for risk model construction and validation in the four cells lines and three OS cell lines, before and after 5-Aza-dC treatment, were detected by qPCR analysis, respectively. The cDNA was synthesized using primeScript RT Master Mix (Perfect Real Time) (Cat. no. RR036A; Takara). PCR was then conducted using Power SYBR Green PCR Master Mix (Cat. no. A25742; Thermo Scientific, MA, USA). GAPDH was applied as the internal control, and all primers sequence are presented in Table 1. The relative expression of the genes was calculated by the 2^−ΔΔCt^ method.

**Table 1:** The primer sequence for each detected gene

Primer name	Primer sequence (5′-3′)
FES-hF-1	TCCCCCTATCCCAACCTCAG
FES-hR-1	ACTGCTCCATGAGCCTGAAC
FYN-hF	TGGAGGTGTGAACTCTTCGTC
FYN-hR	TCTGTCCGTGCTTCATAGTCA
LYL1-hF	ACAGTGTCTACATTGGGCCAG
LYL1-hR	GGCTGCTAGGGAAGATGCT
MAP4K1-hF	TACAGCCACCGCTCTTTGATG
MAP4K1-hR	TGCCTTTTTCCTTCAGTCGGG
RIPK3-hF	ATGTCGTGCGTCAAGTTATGG
RIPK3-hR	CGTAGCCCCACTTCCTATGTTG
SLC15A3-hF	CGGCCAGAGACCGTCAATG
SLC15A3-hR	CACCTGGAAGTTGGCGATG
STAT3-hF	CAGCAGCTTGACACACGGTA
STAT3-hR	AAACACCAAAGTGGCATGTGA
GAPDH-hF	TGACAACTTTGGTATCGTGGAAGG
GAPDH-hR	AGGCAGGGATGATGTTCTGGAGAG

### Lentivirus vectors construction and infection

Full length FES cDNA was amplified by PCR and subcloned into pCDH-CMV-MCS-EF1-copGFP-T2A-Puro lentiviral expression vectors, followed by digestion with Xba I/EcoR I. The recombinant FES overexpression constructs were then transfected into *E. coli* and colonies were cultured in medium containing penbritin. To confirm transfection, PCR and western blot (WB) analysis of individual colonies, and DNA sequencing of recombinant FES overexpression plasmids were performed.

### Cell proliferation and cell cycle assays

The MNNG/HOS cells transfected with FES overexpression plasmid, or empty vector (negative control), as well as normal MNNG/HOS cells (blank) were digested using 0.25% trypsin and centrifuged at 1500 rpm for 5 min. The cells were then seeded into 6-well culture plates and cultured in complete medium at 37 °C with 5% CO_2_. Cell viability was measured after 24, 48, and 72 h post transfection using the Cell Counting Kit-8 (CCK-8, C0039; Beyotime Biotechnology, Shanghai, China). In brief, 10 μL CCK8 solution (5 mg/mL) was added, and the absorbance of each well at 450 nm was measured using a microplate reader (Infinite M100 PRO; TECAN), followed by dark incubation for 2 h.

For cell cycle assays, at 48 h post transfection, the cells were collected through centrifugation at 300*g* for 3 min and washed once with PBS. Afterward, the cells were fixed overnight by adding 6 mL 70% pre-cooled ethanol at 4 °C. Cells were then washed twice with PBS and resuspended in 100 μL binding buffer with adding 50 μg/mL RNase A. Following staining with 5 μL propidium iodide (PI, 50 μg/mL) at 37 °C for 30 min, the percentage of cells in the G1, S, and G2/M phases was detected by flow cytometer (Calibur, BD) and Modfit software was used to analyze the data.

### Cell migration and invasion assays

The effects of FES overexpression on the migration and invasion capacity in MNNG/HOS cells were investigated. Cell migration and invasion were detected using Transwell chambers with polycarbonic membranes (8 mm pore filter size). For the migration assay, cells (100 µL) transfected with FES plasmid or empty vector (3 × 10^6^/mL) in serum-free culture medium were seeded into the upper chamber, while complete medium with 20% FBS (500 µL) was added to lower chamber. After 48 h of incubation at 37 °C, nonmigrated cells were scraped and migrated cells were stained with Crystal Violet. Stained cells were then observed using an Olympus IX73 microscope in six randomized fields (×100). For the invasion assay, the procedures were the same, except 100 μL matrigel (Cat. no. 356234; Corning), diluted with basal medium without serum, was added to the upper chamber.

### Western blot analysis

FYN and β-catenin are reported to be responsible for the development and progression of OS [16, 17]. Interesting, they were predicted to directly or indirectly interact with FES using the STRING database (https://string-db.org/). However, the relationships between FYN, β-catenin, and FES in OS have not been evaluated. Thus, the possibility that overexpressed FES influences the protein expression of FYN and β-catenin was raised. The collected cells in each group were dissolved by RIPA lysis buffer (Cat. no. P0013B; Beyotime), and the supernatants were reserved following centrifugation (11,000*g* at 4 °C for 10 min). The concentration of proteins in each sample was detected using the BCA Protein Assay Kit (Cat. no. PL212989; Thermo Scientific), and then the lysates were separated on 12% SDS-polyacrylamide gels. The separated proteins were transferred into PVDF membrane (Cat. no. IPVH00010; Millipore) and the membranes were incubated with primary antibodies at 4 °C overnight. Following, the membranes were incubated with anti-rabbit or anti-mouse horseradish peroxidase-conjugated second antibodies. The blots were viewed by the Millipore ECL system. β-actin was applied as an internal control. The primary antibodies were rabbit anti-FYN (Cat. no. WL01300; Wanleibio), rabbit anti-β-catenin (D10A8) XP^®^ (Cat. no. 8480S; CST), mouse anti-Flag Tag (Cat. no. 66008-3-Ig, Proteintech), and mouse anti-β-actin (Cat. no. INC Sc-130065, Santa Cruz Biotechnology).

### Statistical analysis

Results were presented as mean ± SD. All statistical analyses were performed using Graphpad Prism 5. The comparisons of quantitative data between control and experimental groups were analyzed by the Student’s t-test, and the multiple comparisons were analyzed using a two-way analysis of variance (ANOVA). A p-value < 0.05 was considered statistically significant.

## Results

### DMP screening and correlation analysis between DMPs and genes

After probe filtration, a total of 26,569 probes were obtained. Then, based on the above screening threshold, a set of 501 DMPs across the OS and normal samples were screened, including 495 hyper-methylated positions and 6 hypo-methylated positions.

In addition, the probes in GSE36001 were totally annotated into 19,569 genes, and above 501 DMPs were matched to 401 genes. The correlation analysis showed that only 130 DMPs were significantly negatively correlated with 114 genes, and these genes were considered candidate genes for future analysis.

### Screening of prognosis relevant genes

Based on the expression values and prognosis relevant clinical information of sarcoma samples provided in the UCSC database, a total of 31 candidate genes corresponding to 36 DMPs were identified as prognosis relevant genes though univariate Cox regression analysis.

### Risk model construction and validation

**Fig. 1:**
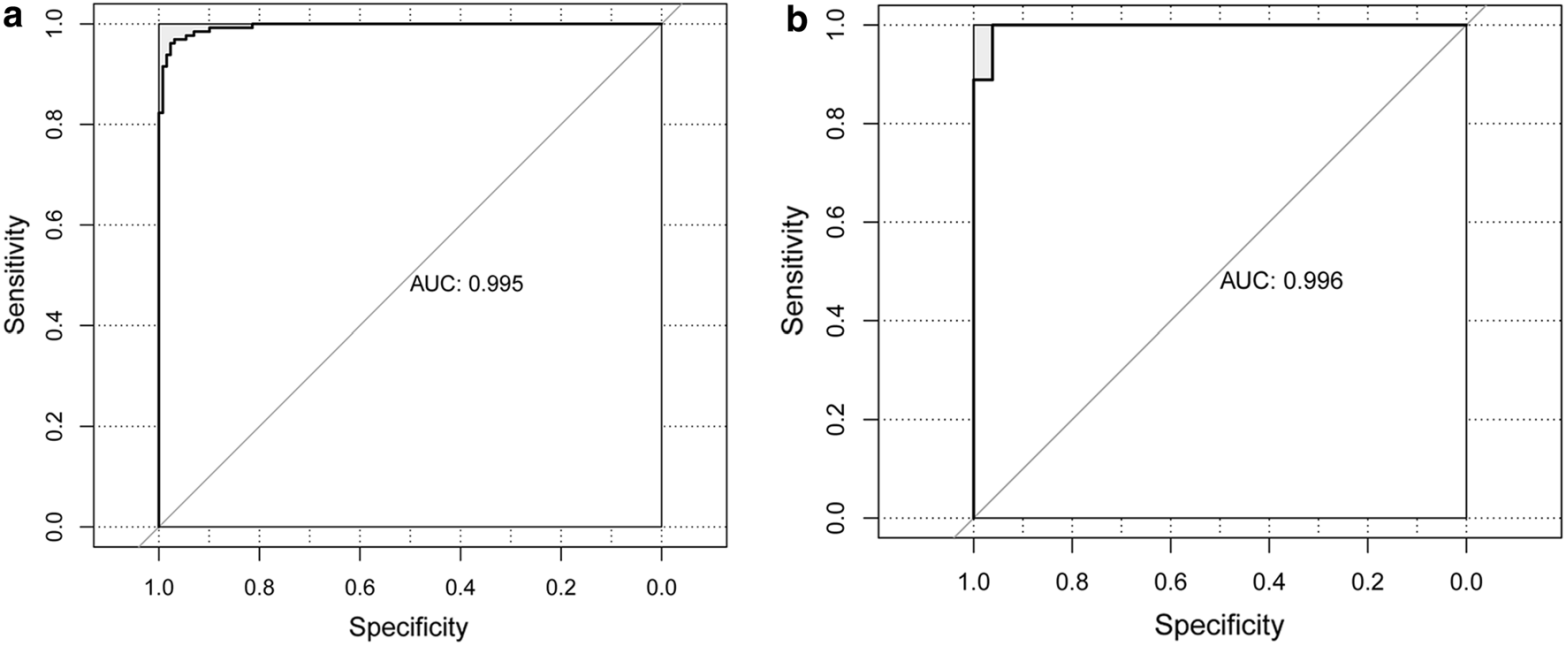
The receiver operating characteristic curves for the training set (**a**) and validation set (**b**)

**Fig. 2:**
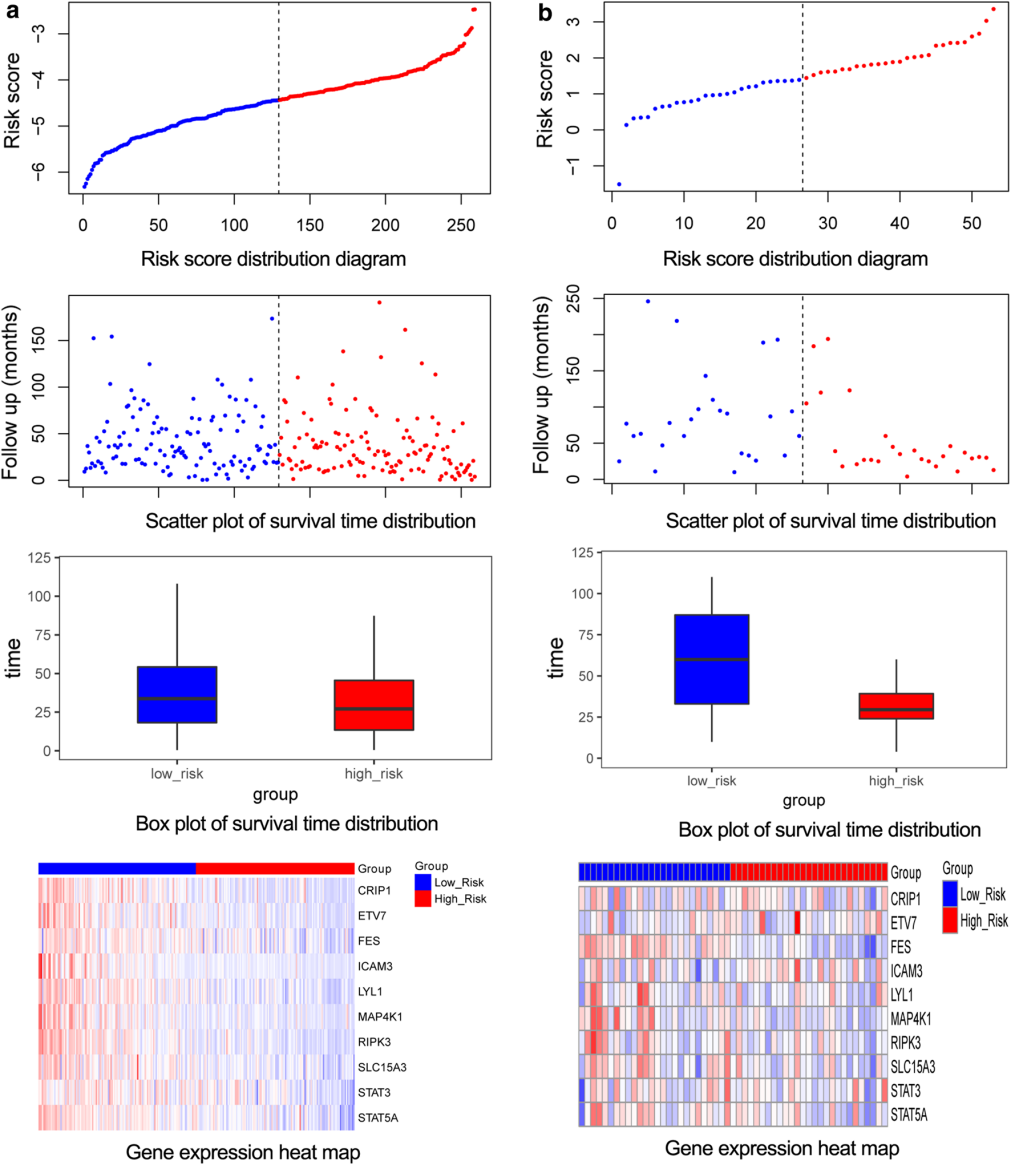
The risk score, survival time, and gene value distribution of high and low risk samples for the training set (**a**) and validation set (**b**). The figures from top to bottom are the risk score distribution, scatter plot of survival time distribution, box plot of survival time distribution, and gene expression heat map, respectively. Red represents the high risk samples, while the blue represents the low risk samples. The color changes of red-white-blue represent the gene expression value from high to low. The genes with higher expression values in low risk samples, and lower expression values in high risk samples may induce a higher risk score

In order to screen the key genes, the top 10 prognosis relevant genes showing hypermethylation were chosen for risk score calculation. When the 10 genes were added to the risk score formula (Eq. 1), the risk score model reached the maximum significant correlation between survival, and high and low risk samples (p = 0.000357612, Table 2), and the AUC value for distinguishing high and low risk samples was 0.995 (Fig. 1a). Thus, 10 genes (RIPK3, STAT5A, LYL1, CRIP1, ICAM3, ETV7, FES, MAP4K1, SLC15A3, and STAT3) were finally used for risk model construction. The risk score distribution revealed that the risk was proportional with risk score, and high-risk samples had a higher risk score than low risk samples (Fig. 2a). In addition, the patients in the high-risk group had shorter survival times than those in the low risk group. Moreover, with the decrease in gene expression, the risk score improved relatively (Fig. 2a).

**Table 2:** The 10 genes prognosis relevant genes used for risk model construction

Gene	p	roc	HR	p value
RIPK3	0.000496906	1	0.508219103	0.000155969
STAT5A	0.006575353	1	0.549351768	0.000284081
LYL1	0.001610084	0.999701849	0.639572451	0.000741679
CRIP1	0.002200324	0.999642218	0.44753046	0.003005436
ICAM3	0.002828331	0.998926655	0.393200464	0.003695443
ETV7	0.000933371	0.999344067	0.673569092	0.004239205
FES	0.004138822	0.997674419	0.661430993	0.004372685
MAP4K1	0.009636057	0.993202147	0.679925064	0.005188387
SLC15A3	0.000583641	0.994514013	0.737447977	0.005444632
STAT3	0.000357612	0.994991055	0.609292291	0.005893217

p indicates the significance between the high and low risk samples and survival, when its corresponding gene is added into risk score model in sequence, while p value indicates the significant correlation between its corresponding gene and prognosis

*ROC* receiver operating characteristic, *HR* hazard ratio

The GSE21257 dataset was used to validate the risk model. The AUC value for high and low risk sample identification by these 10 genes was as much as 0.996 (Fig. 1b). Similarly, it showed that high risk samples had a higher risk score than low risk samples, and the patients in the high-risk group had shorter survival times than those in the low risk group. In addition, with the decrease of gene expression, the total risk score increased relatively (Fig. 2b). In particular, the expression of FES, LYL1, MAP4K1, RIPK3, SLC15A3, and STAT3 varied between different risk samples, showing higher expression values in low risk samples, and lower expression values in high risk samples.

### KM survival analysis

**Fig. 3:**
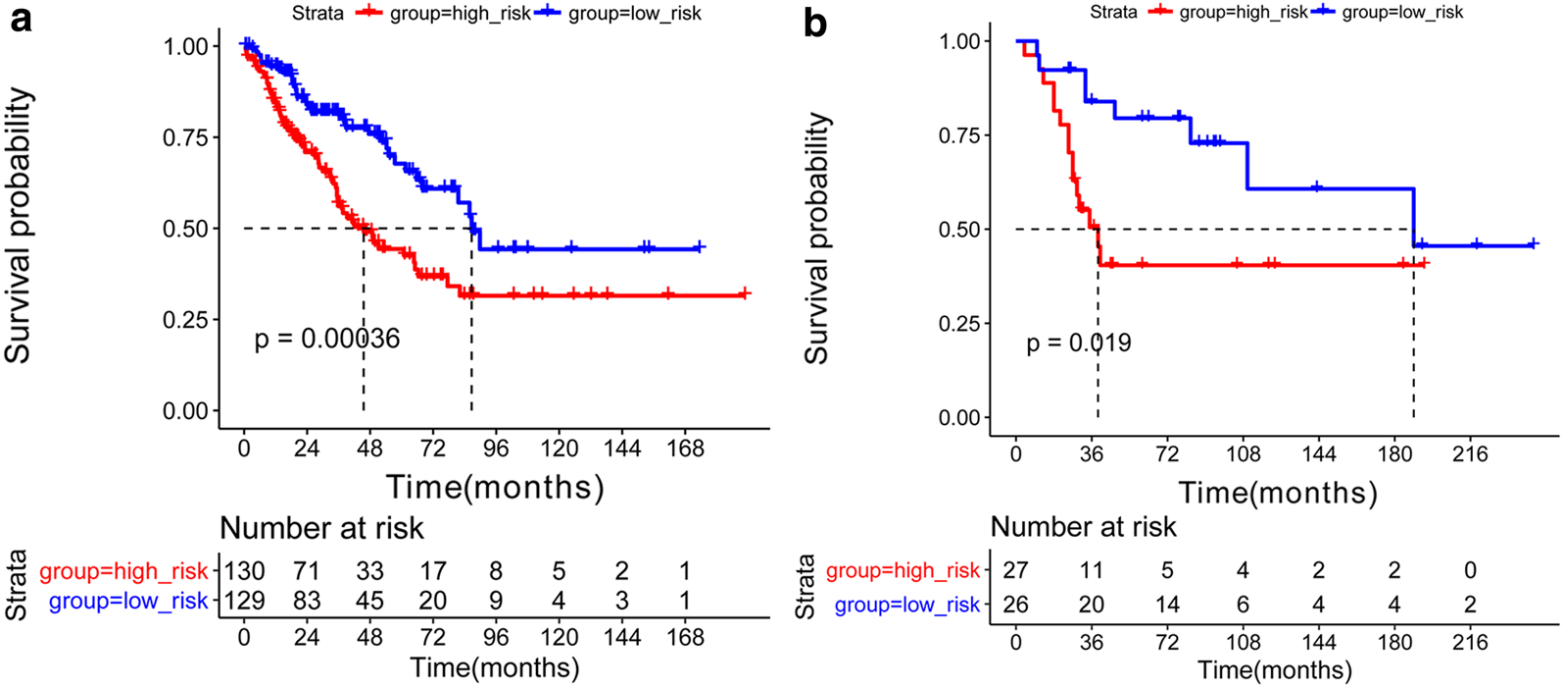
The Kaplan–Meier survival curves of high and low risk groups for the training set (**a**) and validation set (**b**)

According to the risk score model constructed using these 10 genes in the training or validation set, samples were divided into high or low risk groups. For the training set, the result of KM survival analysis showed that there was a significant difference in overall survival time between the high and low risk groups (p = 0.00036, Fig. 3a). This was consistent with the result obtained in the validation set (p = 0.0019, Fig. 3b).

### qPCR verification

**Fig. 4:**
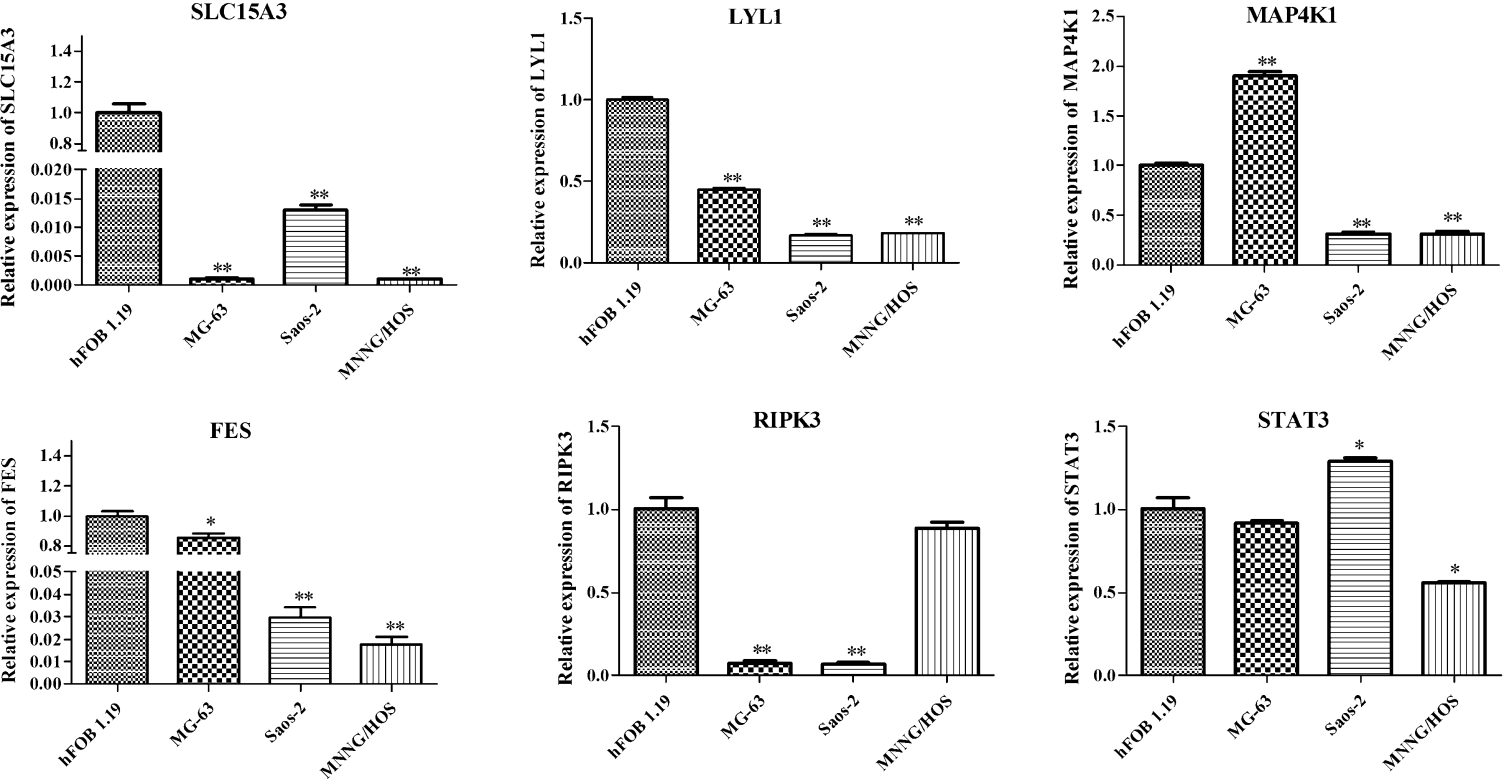
The mRNA levels of SLC15A3, LYL1, MAP4K1, FES, RIPK3, and STAT3 in Saos2, MG63, and MNNG/HOS OS cell lines compared with normal hFOB 1.19 cells, detected by qPCR. *Represents p < 0.05 and ** represents p < 0.01

The six genes (FES, LYL1, MAP4K1, RIPK3, SLC15A3, and STAT3) that showed consistent expression trends in different risk samples, in both the training and validation sets, were selected for further analysis. The qPCR results indicated that expression of SLC15A3, LYL1, and FES were all significantly reduced in the Saos2, MG63, and MNNG/HOS OS cell lines compared with normal hFOB 1.19 cells, while RIPK3 expression was only significantly decreased in the Saos2 and MG63 cell lines (p < 0.01) and no significant differences between MNNG/HOS and hFOB 1.19 were found. Compared with the hFOB 1.19 cell line, the expression of MAP4K1 was remarkably enhanced in the MG63 cell line (p < 0.01), but significantly reduced in the Saos2 and MNNG/HOS cell lines (p < 0.01). In addition, STAT3 was significantly overexpressed in the Saos2 cell line, but decreased in the MNNG/HOS cell line. No significant change in STAT3 was found between the hFOB 1.19 and MG63 cell lines (Fig. 4).

### The expression of key genes after methylase inhibitor treatment

**Fig. 5:**
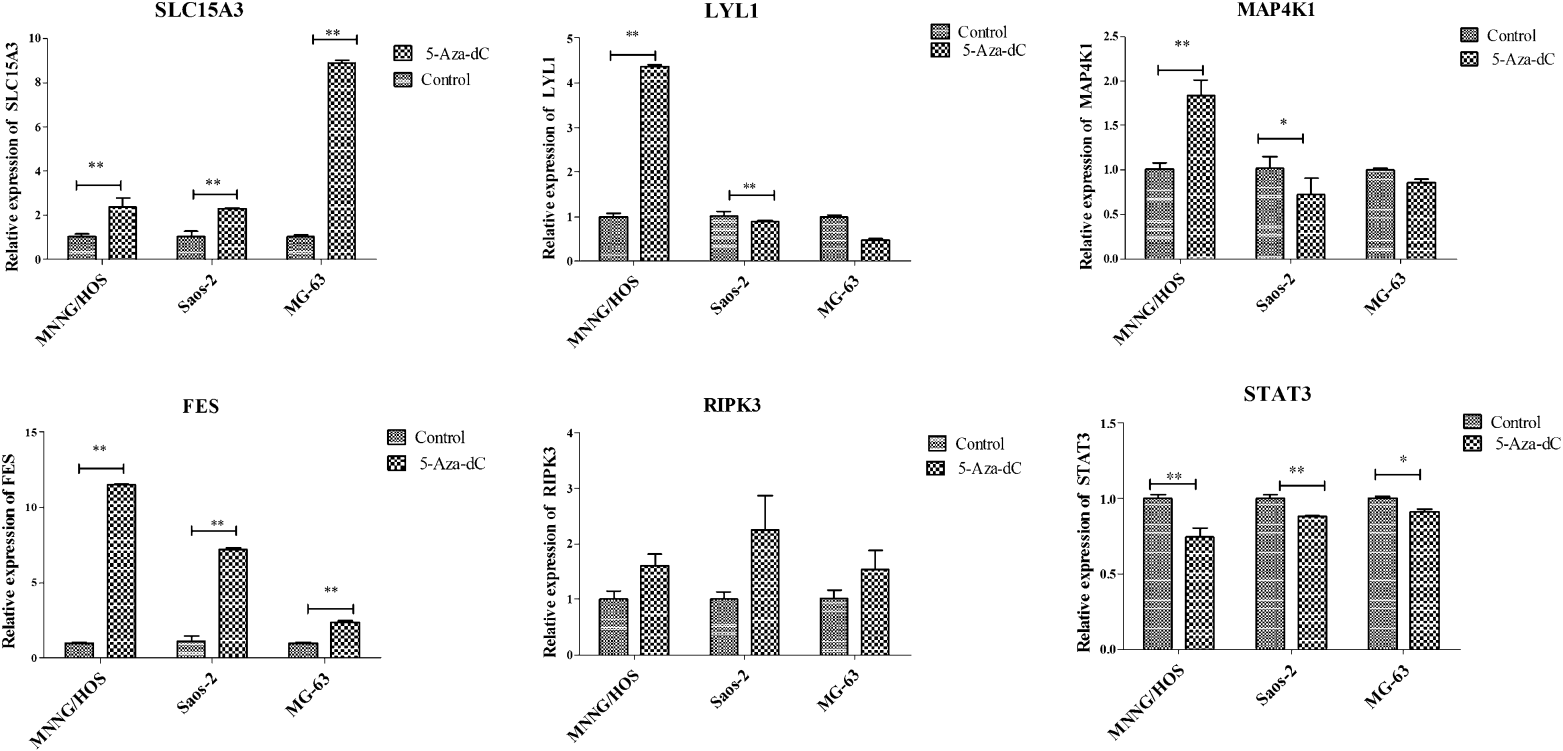
The mRNA levels of SLC15A3, LYL1, MAP4K1, FES, RIPK3, and STAT3 in Saos2, MG63, and MNNG/HOS OS cell lines before and after 5-Aza-dC treatment, detected by qPCR. *Represents p < 0.05 and ** represents p < 0.01

According to bioinformatic analysis, the six key genes were hyper-methylated in OS samples compared with normal samples. We further analyzed whether their high methylation statuses were involved with their expression changes. The results showed that increased expression of FES and SLC15A3 were found in Saos2, MG63, and MNNG/HOS, the three OS cell lines treated with 5-Aza-DC (p < 0.01), whereas after 5-Aza-DC treatment, STAT3 expression was significantly upregulated in the three OS cell lines (p < 0.01). In addition, the expression of LYL1 and MAP4K1 were enhanced in the MNNG/HOS cells, but reduced in MG63 cells treated with 5-Aza-DC. No significant changes in LYL1 and MAP4K1 were found in Saos2 cells. Similarly, no obvious changes in RIPK were detected in the three OS cell lines between pre- and post-5-Aza-DC treatment (Fig. 5).

### FES overexpression inhibits the proliferation, cell cycle progression, migration, and invasion of MNNG/HOS cells

**Fig. 6:**
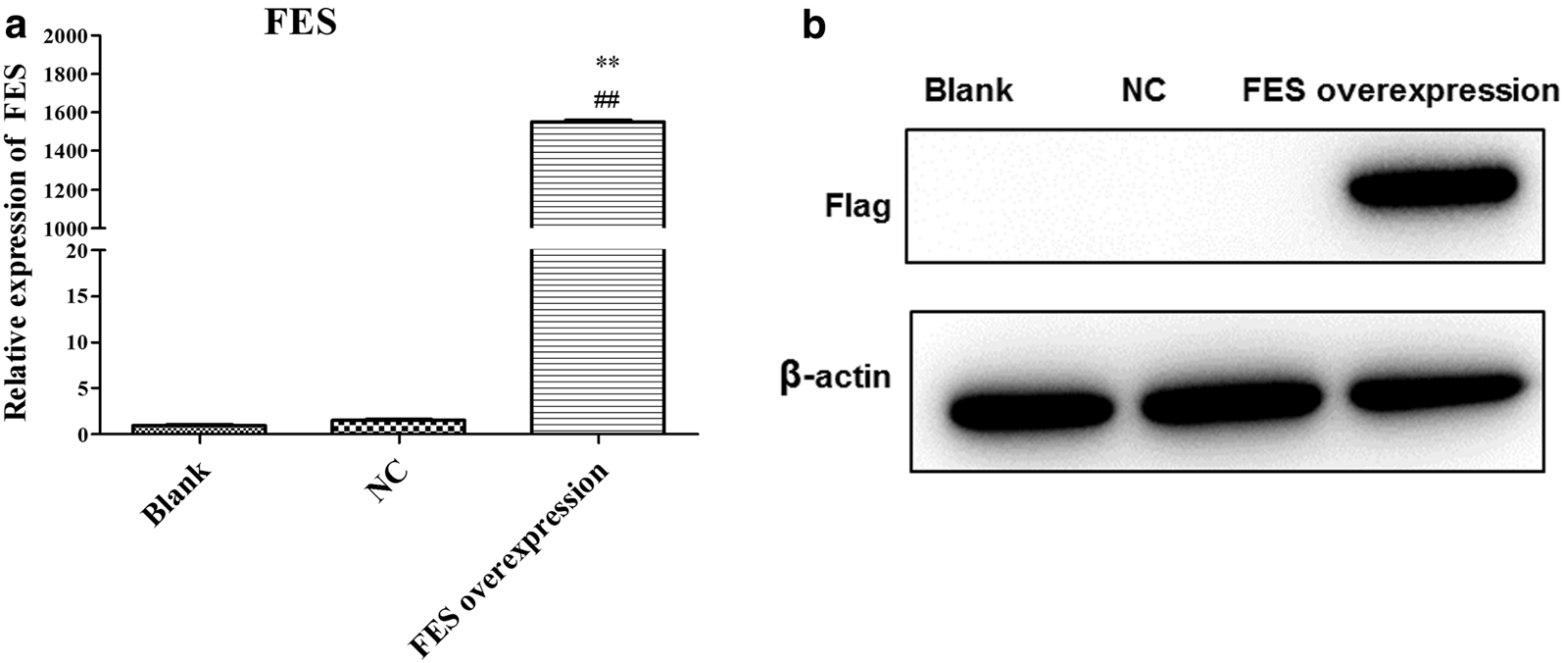
The mRNA (**a**) and proteins levels of FES (**b**) in MNNG/HOS cells transfected with FES overexpression plasmid, detected by qPCR and western blot. **Represents p < 0.01 compared with the NC group, while ## represents p < 0.01 compared with the Blank group

**Fig. 7:**
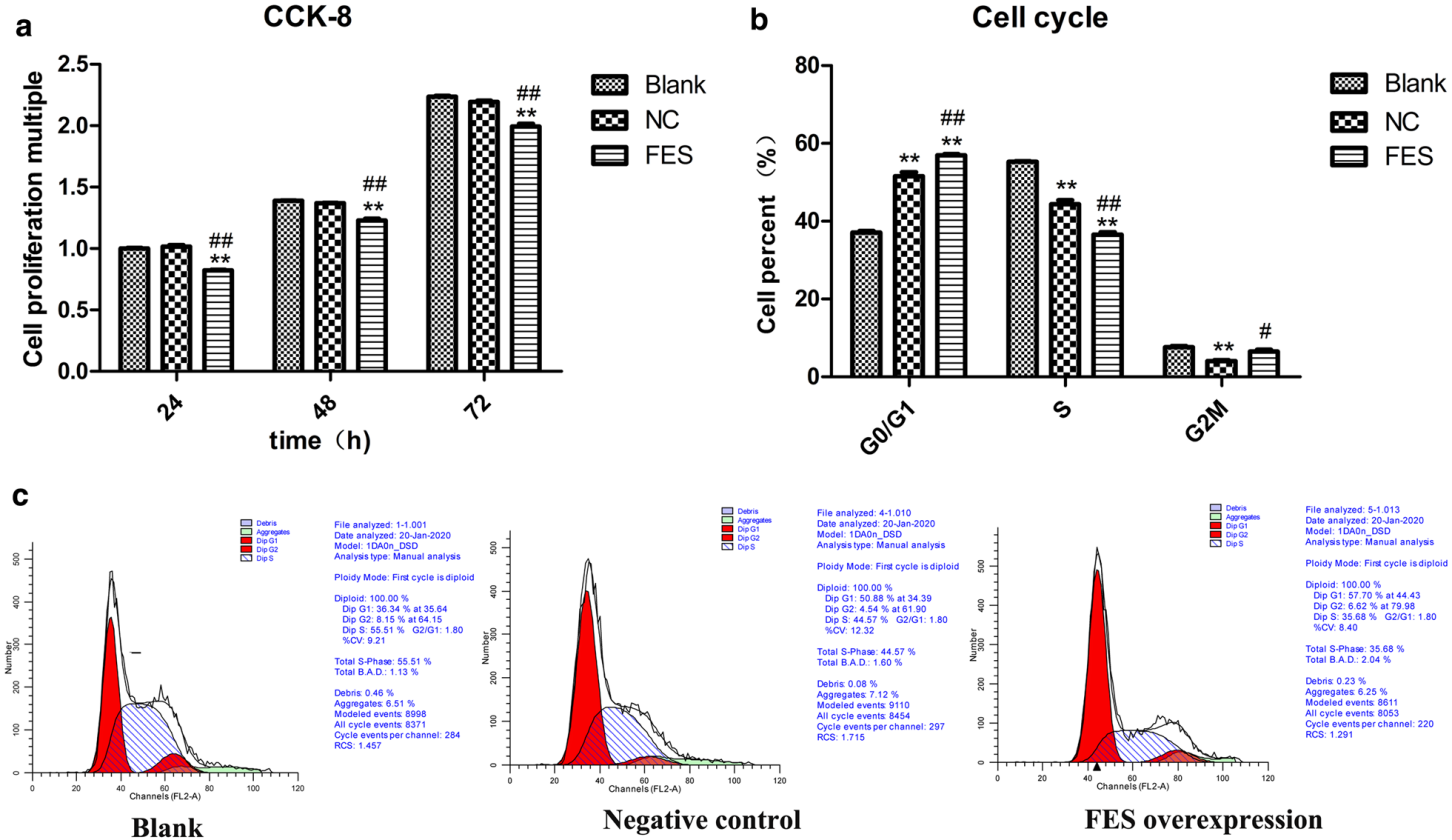
Effect of FES overexpression on cell proliferation at 24, 48 and 72 h post-transfection (**a**) and the percentage of cell cycle distribution in different groups presented by graphs (**b**) and histograms (**c**) in MNNG/HOS cells. *Indicates p < 0.05 and ** represents p < 0.01 compared with the Blank group, while # indicates p < 0.05 and ## represents p < 0.01 compared with the NC group

**Fig. 8:**
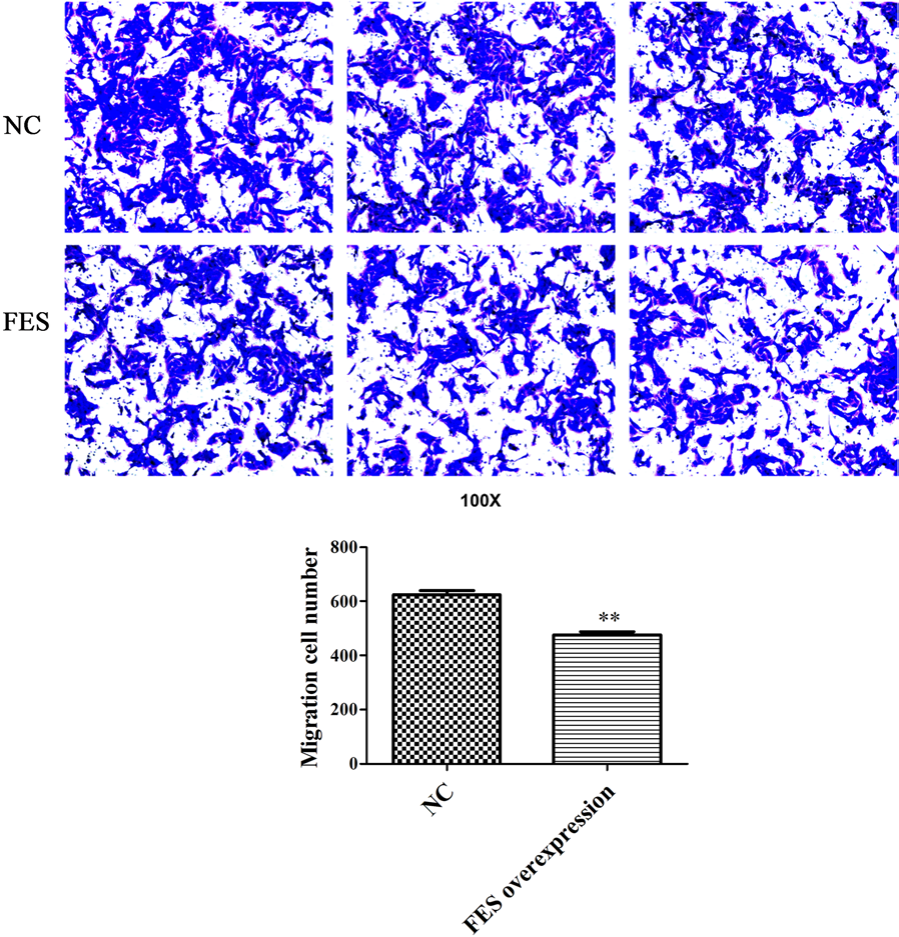
Effect of FES overexpression on cell migration in MNNG/HOS cells. **Represents p < 0.01

**Fig. 9:**
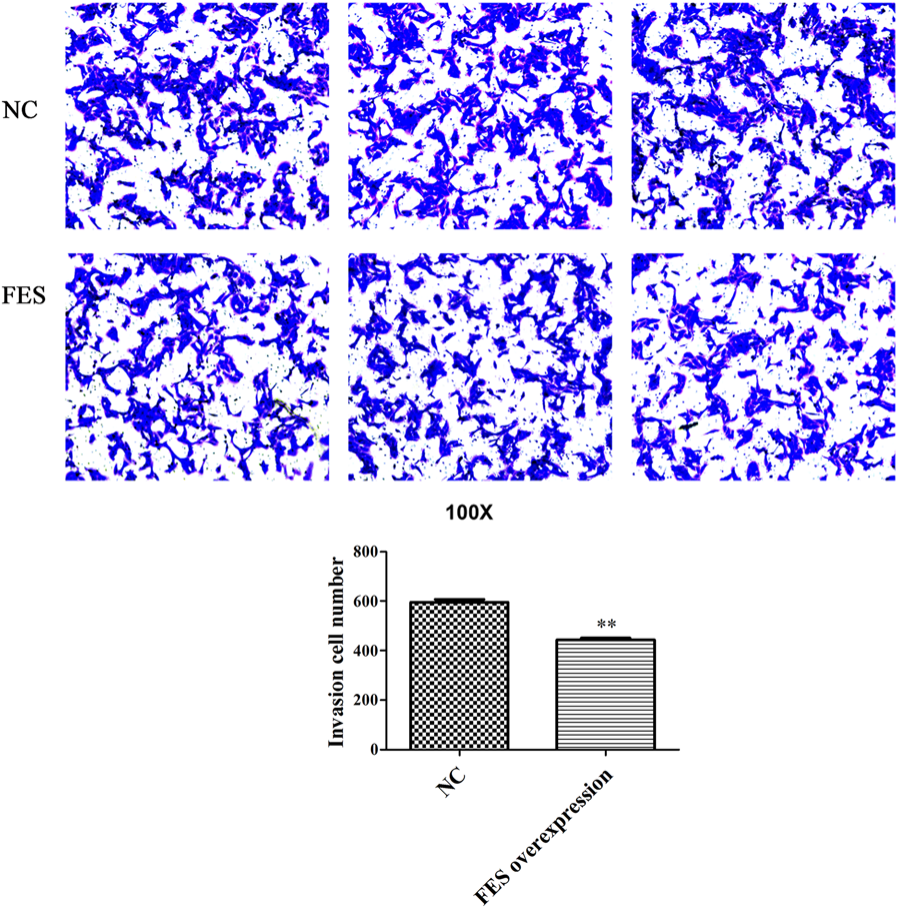
Effect of FES overexpression on cell invasion in MNNG/HOS cells. **Represents p < 0.01

Using qPCR and WB analyses, FES expression was shown to be significantly enhanced in MNNG/HOS cells transfected with FES overexpression plasmid, compared with cells transfected with an empty vector (p < 0.01, Fig. 6a, b), indicating that FES overexpression plasmids were successfully constructed and transfected. The result of the CCK8 assay revealed that FES overexpression was able to significantly reduce the viability of MNNG/HOS cells after 24, 48, and 72 h post-transfection (p < 0.01, Fig. 7a). Additionally, after 48 h post-transfection, FES overexpression significantly blocked MNNG/HOS cells at the G0/G1 phase with a concomitant decrease of cells in the S phase (p < 0.01, Fig. 7b, c). Notably, the overexpression of FES markedly suppressed the migration and invasion activities of MNNG/HOS cells (p < 0.01, Figs. 8 and 9).

### Western blot analysis of FES related genes

**Fig. 10:**
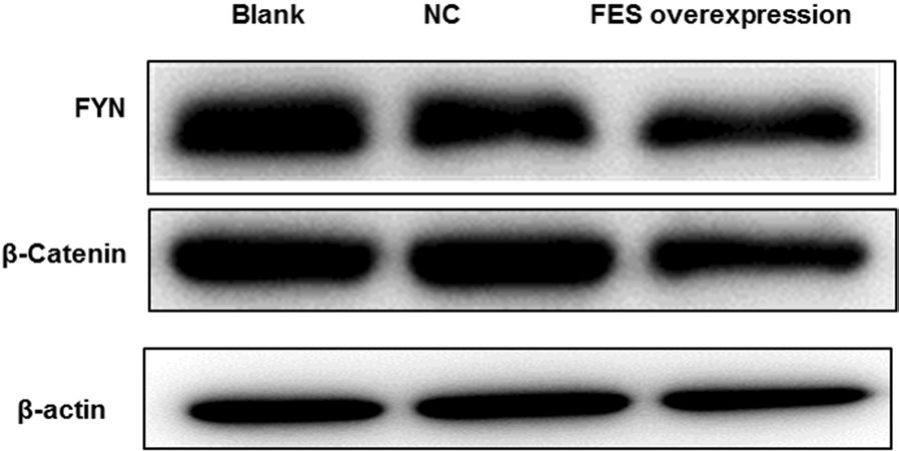
Effect of FES overexpression on protein expression levels of FYN and β-catenin in MNNG/HOS cells

The effects of FES overexpression on FYN and β-catenin expression were explored. As a result, the protein expression of FYN and β-catenin were both significantly reduced in MNNG/HOS cells with FYN overexpression, compared with MNNG/HOS cells or MNNG/HOS cells transfected with empty plasmid (Fig. 10).

## Discussion

In this study, 31 candidate genes, corresponding to 36 DMPs, were identified as OS prognosis relevant genes, among which, the top 10 genes that showed hypermethylation were used for constructing a risk model. After validating the risk model using data from the GSE21257 dataset, FES, LYL1, MAP4K1, RIPK3, SLC15A3, and STAT3 showed expression changes between OS and control samples. Further, qPCR results showed that FES expression was significantly downregulated in the three OS cell lines compared with control cells, and increased after 5-Aza-DC treatment. Additionally, the proliferation, migration, and invasion of OS cells were significantly suppressed after FES overexpression, and the expression of FYN and β catenin were reduced in OS cells transfected with FES overexpression plasmid.

FES Proto-Oncogene Tyrosine Kinase (FES, also known as FPS), is a dominant-acting oncoprotein that has tyrosine-specific protein kinase activity [18]. It contains a central Src homology-2 domain and a COOH-terminal tyrosine kinase catalytic domain [19]. FES was initially identified as an oncogene from the tumor-causing feline sarcoma retrovirus [20]. However, the notion that FES acts as a tumor suppressor in breast epithelial cells has been recently proposed [21]. In our study, FES, predicted as a key OS prognosis gene by the risk score model, was found to be hypermethylated and its expression level was correspondingly downregulated in OS cells. Similarly, Shaffer et al. [22] demonstrated that extensive methylation of the FES promoter contributes to inhibition of FES expression in colorectal cancer cells. In addition, Kuo et al. [23] showed that the high methylation level of FES is a biomarker for prognosis prediction in esophageal squamous cell carcinoma patients, via risk score analysis. In addition, the methylation level of FES is positively related with tumor stage in breast cancer [24]. However, the relationship between FES and OS has not been evaluated. It has been reported that OS is a tyrosine kinase-related disorder [25], and FES, as a cytoplasmic protein tyrosine kinase, is highly expressed in vascular endothelial and myeloid cells [26]. Mitsui et al. [27] found that semaphorin 3A can activate FES and enhance tyrosine phosphorylation of the CRAM–CRMP complex. Notably, the expression of semaphorin 3A was reduced in the MNNG/HOS OS cell line, which is implicated in ectopic bone formation, osteolysis, cell growth, motility, and invasion in OS [28]. Thus, we suggest that the reduced expression of FES induced by its hypermethylation status may be responsible for OS progression and prognosis.

Reportedly, aberrantly activated FES is related to the proliferation, migration, and invasion of several neoplasms [29, 30]. Voisset et al. [29] proposed that FES is an upstream regulator of KIT and participates in the KIT-related proliferation signal process. In addition, downregulated FES can inhibit the proliferation of renal carcinoma cells [30]. Moreover, activation of the PLD2-Fes-Jak3 signaling pathway can accelerate the proliferation and invasion of breast cancer cells [31]. Furthermore, Fes deficiency is reported to reduce the cell adhesion and migration of bone marrow-derived mast cells, via the downstream regulation of Kit and beta 1 integrin receptors [32]. Although the overexpression of FES had been historically considered to promote the proliferation, migration, and invasion of tumor cells due to its proto-oncogene characteristics, our results showed that FES overexpression was able to inhibit the proliferation, migration, and invasion of OS cells. Similarly, it has been suggested that activated FES could suppress the invasion of colon colorectal HCT 116 cells [22]. These opposite functions of FES in different tumor cells may be due to its double-sided roles as a proto-oncogene or tumor suppressor.

Proto-Oncogene C-Fyn (FYN) is also a Src tyrosine kinase family member, which is implicated in the regulation of the cytoskeleton, integrin signaling, and cell growth [33, 34]. Similar to FES, it has been historically considered to have carcinogenic properties. Interesting, Sørensen et al. [35] detected a significant downregulation of FYN in prostate cancer tissue, highlighting its tumor suppressive role. β catenin is a multifunctional protein, and crucial for regulating cell adhesion and the Wnt signaling pathway via its phosphorylation modification [36]. Zhu et al. [37] reported that knockdown of XRCC6 expression can suppress cell proliferation of MNNG/HOS and U2OS OS cell lines via reducing β catenin expression. Reportedly, β catenin functions as a docking protein to facilitate the activation of Fer/Fyn tyrosine kinases [38]. Notably, Fer is reported to have similar biological roles with FES due to the close structural similarity between them [39]. In addition, FYN is also an upstream kinase of FES that facilitates FES activation [40]. In our study, expressions of FYN and β catenin were lower in OS cells after transfection with FES overexpression plasmid. Taken together, FES, regulated by its upstream FYN and β catenin, might coordinately exert a tumor suppressor effect in OS cells.

## Conclusions

The bioinformatic analysis results showed that the risk model constructed by 10 OS prognosis relevant genes could be used for OS prognosis, with high AUC value. The reduced expression of FES, induced by its hypermethylation status, may be responsible for OS progression and prognosis. In addition, the overexpression of FES could inhibit the proliferation, migration, and invasion of OS cells. FES, regulated by its upstream FYN and β catenin, might coordinately exert a tumor suppressor effect in OS cells.

## Data Availability

The dataset supporting the conclusions of this article is included within the article.

## Abbreviations

OS: Osteosarcoma
DMP: Differentially methylated position
5-Aza-dC: 5-Aza-2′-deoxycytidine
FC: Fold change
KM: Kaplan–Meier
FBS: Fetal bovine serum
qPCR: Quantitative polymerase chain reaction
